# How Effective Is a Traffic Control Policy in Blocking the Spread of COVID-19? A Case Study of Changsha, China

**DOI:** 10.3390/ijerph19137884

**Published:** 2022-06-27

**Authors:** Wang Xiang, Li Chen, Qunjie Peng, Bing Wang, Xiaobing Liu

**Affiliations:** 1 Key Laboratory of Special Environment Road Engineering of Hunan Province, Changsha University of Science & Technology, Changsha 410114, China; xiangwang@csust.edu.cn (W.X.); chenlijiaotong@stu.csust.edu.cn (L.C.); 2 Shenzhen Transportation Design & Research Institute Co., Ltd., Shenzhen 518003, China; baxiayy@163.com; 3 Changsha Planning & Design Survey Research Institute, Changsha 410007, China; amberslience@163.com; 4 Key Laboratory of Transport Industry of Big Data Application Technologies for Comprehensive Transport, Beijing Jiaotong University, Beijing 100044, China

**Keywords:** COVID-19, urban epidemic control, traffic control policy, infectious disease model

## Abstract

(1) Background: COVID-19 is still affecting people’s daily lives. In the past two years of epidemic control, a traffic control policy has been an important way to block the spread of the epidemic. (2) Objectives: To delve into the blocking effects of different traffic control policies on COVID-19 transmission. (3) Methods: Based on the classical SIR model, this paper designs and improves the coefficient of the infectious rate, and it builds a quantitative SEIR model that considers the infectivity of the exposed for traffic control policies. Taking Changsha, a typical city of epidemic prevention and control, as a study case, this paper simulates the epidemic trends under three traffic control policies adopted in Changsha: home quarantine, road traffic control, and public transport suspension. Meanwhile, to explore the time sensitivity of all traffic control policies, this paper sets four distinct scenarios where the traffic control policies were implemented at the first medical case, delayed by 3, 5, and 7 days, respectively. (4) Results: The implementation of the traffic control policies has decreased the peak value of the population of the infective in Changsha by 66.03%, and it has delayed the peak period by 58 days; with the home-quarantine policy, the road traffic control policy, and the public transport suspension policy decreasing the peak value of the population of the infective by 56.81%, 39.72%, and 45.31% and delaying the peak period by 31, 18, and 21 days, respectively; in the four scenarios where the traffic control policies had been implemented at the first medical case, delayed by 3, 5, and 7 days, respectively, the variations of both the peak value and the peak period timespan of confirmed cases under the home-quarantine policy would have been greater than under the road traffic control and the public transport suspension policies. (5) Conclusions: The implementation of traffic control policies is significantly effective in blocking the epidemic across the city of Changsha. The home-quarantine policy has the highest time sensitivity: the earlier this policy is implemented, the more significant its blocking effect on the spread of the epidemic.

## 1. Introduction

Over the last three years, the whole world has been involved in the fight against COVID-19 [1]. Having mutated from the initial culprit of the original symptoms to the asymptomatic Delta variant in 2021 to the current Omicron variant with extremely strong infectivity [2,3], COVID-19 has brought about unprecedented physical injuries and mental traumas to human beings [4,5]. Despite huge progress in medical science and epidemic prevention and control [6,7], the daily number of global cases goes up and up by millions in a day. As of April 2022, the total number of confirmed cases had reached 505 million, with a total death toll of 6.21 million people worldwide [8].

Traffic control has had a key role in blocking the spread of the epidemic [9,10] by the main principle of diminishing population aggregation and mobility [11,12]. To block the development of the epidemic, most countries have adopted traffic control policies to varying degrees. The kind of traffic control policies adopted, the effectiveness of controls, and the date of implementation have all varied as a result of the epidemic’s characteristics and differences [13,14,15]. Therefore, studying the effectiveness and the practicality of different traffic control policies in blocking citywide epidemic spread is vitally significant to citywide epidemic prevention and control.

In the existing research, the methods for evaluation of the effectiveness of COVID-19 prevention and control policies are generally divided into two classes: The first class are classical epidemic models typified by SI and SIR [16,17], on the basis of which many scholars have put forward a series of variant models such as SIRQ, SEAHIR, and SEIQR models according to the characteristics of COVID-19. Scholars have studied the separate effectiveness of such prevention and control measures as public health governance, social distancing, and vaccination using these models [18,19,20]. This class of models can clearly and effectively describe the epidemic development of COVID-19 in different situations, and they can predict the peak value and the peak period of the epidemic for decision-makers. However, they are ill-designed in the parameter of the infectious rate and ill-considered in factors of how traffic control policies influence epidemic development. The other class of methods are predominated by generalized linear regression analyses. These models usually set policy factors and evaluate the effectiveness of the control policy according to the effect values of such policy factors through regressive calculation of panel data [21,22,23]. Unfortunately, this class of methods are neither able to simulate the big picture of epidemic development nor able to predict the peak period and the peak value of the epidemic. The relevant work they have done is detailed in Table 1. From previous studies, it is concluded that researchers set up more populations to study the policy effect or the evolution of the epidemic situation in the traditional room-to-room model of infectious diseases, but they lack the research to discuss the policy effect from the angle of infection rate design. The purpose of this paper is to propose a model for evaluating the effects of policies designed from infection rates.

**Figure 1:**
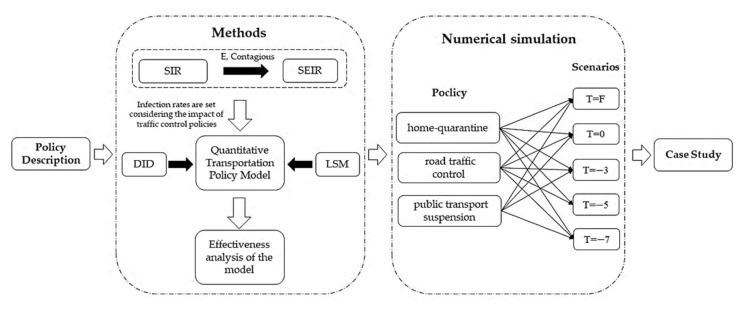
The research method diagram.

The research method diagram of this paper is shown in Figure 1. Considering the infectivity of the exposed for traffic control policies, this paper develops a quantitative SEIR model out of the classical SIR model by giving mere consideration to the infectivity of the exposed in pertinence to the characteristics of the population involved with COVID-19 and by designing and improving the coefficient of the infectious rate and introducing the parameter of traffic control policy effect. Taking Changsha as the study case, based on the big data from Baidu Qianxi, this paper utilizes the empirical model DID (Difference-in-Difference model) to calibrate the policy effect parameter of this city. Finally, it evaluates the effectiveness and the practicality of the three traffic control policies implemented in Changsha: home quarantine, road traffic control, and public transport suspension. The study can provide technical reference for urban traffic control policymaking in the face of similar public health emergencies.

This paper is structured as follows: Section 2 introduces the relevant materials needed for the study; Section 3 introduces the COVID-19 spreading models; Section 4 presents the results; and Section 5 offers a discussion, and it concludes the paper.

## 2. Materials

### 2.1. Regional Overview and Traffic Control Policies

Changsha, the capital of Hunan Province, is only 279 km from Wuhan, which is the epicenter of the 2020 epidemic. Changsha was under massive pressure to prevent and to control the epidemic. Fortunately, Changsha never suffered from any massive outbreak of the epidemic in 2020, and it has achieved a good effect of prevention and control. Therefore, the study on the traffic control policies in Changsha can be enlightening and of exemplary significance to some extent.

On 24 January 2020, Changsha started its first-level public health emergency response. Following that, the Changsha municipality headquarters for COVID-19 prevention and control raised the alarm to citizens urging them to avoid unnecessary outings and keep quarantined at home. On 27 January, the Changsha Transportation Bureau set epidemic prevention and control points at all entrances to Changsha for health code checks, supervision over disinfection and protection, and traffic control of expelling intruders from high- and medium-risk areas and only letting in and not letting out. On 28 January, some bus/coach routes went out of service in the high- and medium-risk areas of Changsha, where the metros were subjected to a skip-station operation. See Table 2 for the key time nodes and implementation ranges.

### 2.2. Dataset

**Figure 2:**
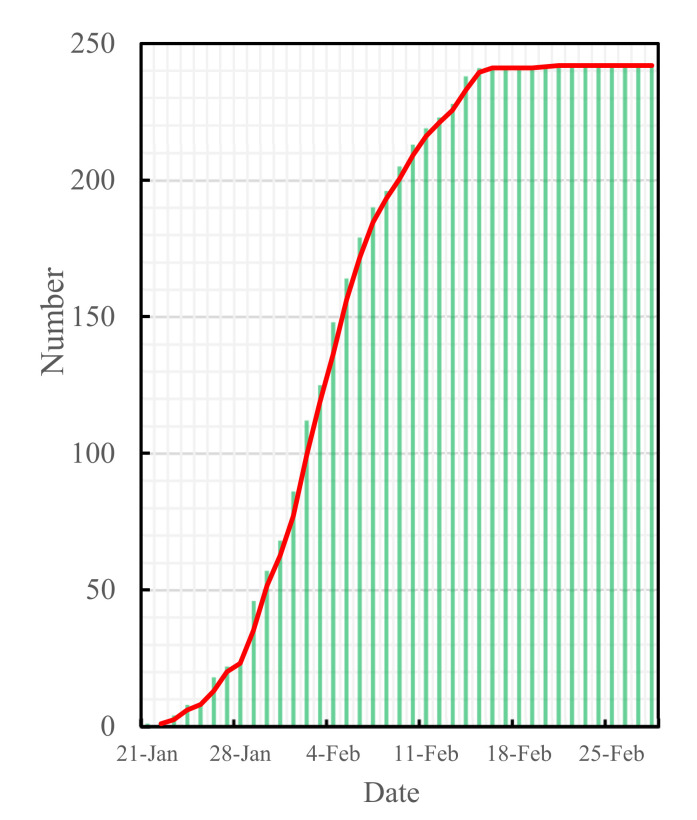
Cumulative confirmed cases in Changsha.

**Figure 3:**
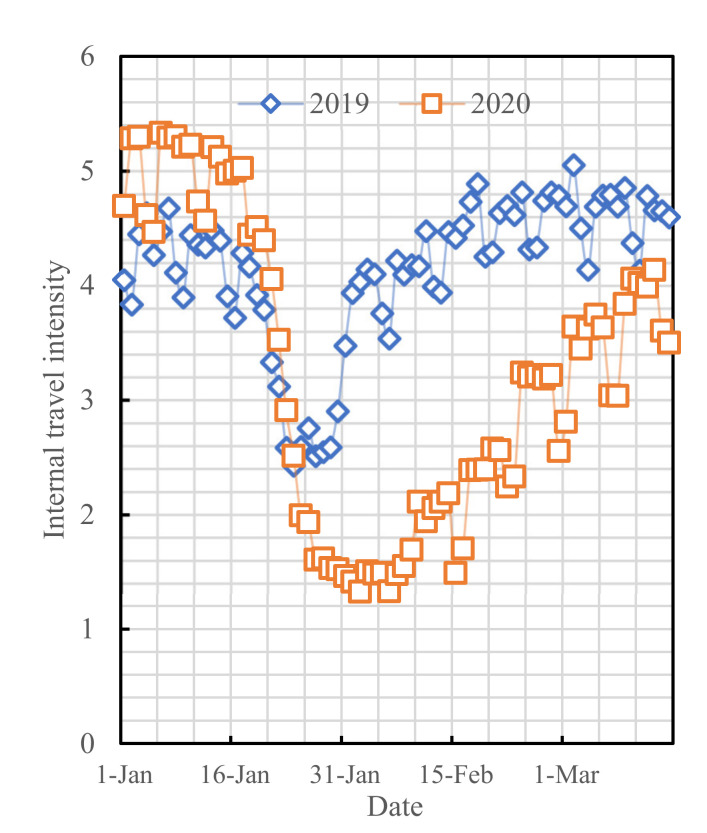
Intracity activity intensities within Changsha in the same period of 2020 versus 2019.

The data of COVID-19 cases used in this study originated from the Changsha Health Commission [24], and they were collected during the period of 21 January to 28 February 2020, as shown in Figure 2. This study also used the big data from the Baidu Qianxi website (http://qianxi.baidu.com/, accessed on 12 February 2021), which was collected during the period of 1 January to 15 March 2020, as shown in Figure 3:

## 3. Methods

SIR is a classical epidemic dynamic model which can effectively simulate the generation, development, propagation, and elimination of epidemics. The SIR model usually sets the study area as an enclosed compartment, and it divides the population therein into three types: the susceptible (S) subpopulation, the infective (I) subpopulation, and the removed (R) subpopulation. Parameters such as the infectious rate and the cure rate are set in this model, and they are subject to state transition from one subpopulation to another according to certain rules [16]. Considering the difference of COVID-19 from traditional epidemics that it has a certain latent period, improvements were made on the base of the SIR model by including the exposed (E) subpopulation for a more reasonable simulation of COVID-19 development.

### 3.1. Construction of the SEIR Model Considering the Infectivity of the Exposed

This model is based upon the following definitions and hypotheses:

- that the susceptible (S) subpopulation is a healthy population that has never been infected with the virus and that is non-immune;
- that the exposed (E) subpopulation is a population that carries the virus and that has an infective capacity during the latent period;
- that all of the infective (I) subpopulation are cured or quarantined immediately after being confirmed and lose infectivity;
- that the recovered (R) subpopulation would not be reinfected after being cured;
- that for the total population size N, mere consideration is given to the effective size of the active population rather than to the births and the deaths within the population and to immigration into and emigration from the population, with all the time;
- that in the early stage of the epidemic, the cumulative confirmed cases are new cases in a single day, whereas the cumulative cured cases are new cured cases in a single day.

**Figure 4:**
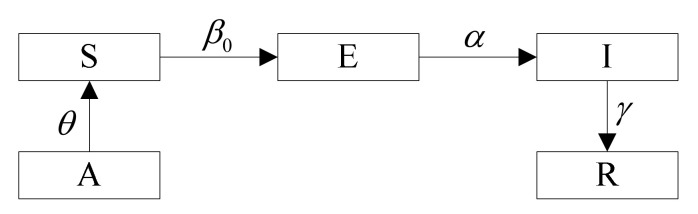
Model Definitions. A is the population that usually lives in the urban area of Changsha, and the proportion of the active population in the urban area is θ. This population constitutes S. Due to COVID-19 having an exposed period, S has a β probability of being converted to E after contact with population E. In E, there is a α probability of being converted to I by nucleic acid detection. Traffic control policies can cut off the contact between S and E, which in turn reduces the infection rate of E.

A system of differential equations is set up as shown in Equations (1)–(5), and the model definitions is shown in Figure 4:

(1) $$\frac{dS(t)}{dt}=-\frac{r\beta_{0}E(t)S(t)}{N}$$

(2) $$\frac{dE(t)}{dt}=\frac{\beta_{0}E(t)S(t)}{N}-\alpha E(t)$$

(3) $$\frac{dI(t)}{dt}=\alpha E(t)-\gamma I(t)$$

(4) $$\frac{dR(t)}{dt}=\gamma I(t)$$

(5) N(t)=S(t)+E(t)+I(t)+R(t)

All parameters are explained in Table 3.

### 3.2. Traffic Control Policy Evaluation Model Based on the SEIR Model Considering the Infectivity of the Exposed

Traffic control policies reduce the infectious rate by decreasing the average number of people to whom the exposed are exposed, thereby reaching the goal of containing epidemic spread. After the outbreak of COVID-19, traffic control policies would have an exponential-scale effect on the variation of population mobility intensity [25,26]. This paper assumes that traffic control policymaking can usually have an exponential-scale effect on the transmission of COVID-19 cases. The parameter of the infectious rate is set as Equation (6):

(6) $$\beta (x)=\beta_{0}\times e^{Kx(t,g,s)}$$

where x(t,g,s) indicates the prevention and the control effort of the traffic policy in question is a function of the promulgation time (*t*), administrative grade of implementation (*g*), and control sphere (*s*); *K* is a policy effect parameter; $\beta_{0}$ is the initial infectious rate. The quantitative model for traffic control policies is shown as Equations (7)–(11):

(7) $$S(t+1)=S(t)-r\frac{\beta_{0}e^{kx(t,g,s)}E(t)S(t)}{N}$$

(8) $$E(t+1)=E(t)+r\frac{\beta_{0}e^{kx(t,g,s)}E(t)S(t)}{N}-\alpha E(t)$$

(9) I(t+1)=I(t)+αE(t)−γI(t)

(10) R(t+1)=R(t)+γI(t)

(11) N(t)=S(t)+E(t)+I(t)+R(t)

See Table 3 for the connotations of parameters.

### 3.3. Parameter Calibration Models

#### 3.3.1. Calibration Model on Initial Infectious Rate $\beta_{0}$ and Cure Rate γ

This paper selects the least square method (LSM) to calibrate the initial infectious rate $\beta_{0}$ and the cure rate γ, taking the parameters with the least sum of squared errors between the actual values of LSM and the predicted values as the optimal solutions. Transforming Equation (7) gives:

(12) $$S(t+1)-S(t)=-r\frac{\beta_{0}e^{kx(t,g,s)}E(t)S(t)}{N}$$

Let S(t+1)−S(t)=Y, $-r\frac{e^{kx(t,g,s)}E(t)S(t)}{N}=X$, and assume $\varphi ={\sum_{t=1}^{m}\left(Y_{t}-\bar{y}\right)}^{2}$. When φ takes the minimum, take the derivative of $\beta_{0}$ to give:

(13) $$\frac{d\varphi }{d\beta_{0}}=2\sum_{t=1}^{m}(\beta_{0}X_{t}{}^{2}-Y_{t}X_{t})$$

Let $\frac{d\varphi }{d\beta_{0}}=0$. Solve for the infectious rate $\beta_{0}$ as expressed in Equation (14):

(14) $$\beta_{0}=\frac{N\sum_{t=1}^{m}\left[S(t+1)-S(t)\right]}{-re^{kx(t,g,s)}\sum_{t=1}^{m}E(t)S(t)}$$

Likewise, solve for the cure rate γ as expressed in Equation (15):

(15) $$\gamma =\frac{\sum_{t=1}^{m}\left[R(t+1)-R(t)\right]}{\sum_{t=1}^{m}I(t)}$$

#### 3.3.2. Calibration Model on Policy Effect Parameter *K*

The policy effect parameter *K* reflects the effect of implementing traffic control policies. This paper selects the empirical DID Model, which is mostly used in econometrics for public policy or program implementation effect, to calibrate the policy effect parameter. The design of this model is formulated by Equation (16):

(16) $$\ln(S_{i,t})=\omega_{0}+\omega_{1}\cdot R_{i,t}+K\cdot T_{i,t}\cdot R_{i,t}+\omega_{3}\cdot T_{i,t}+\varepsilon_{i,t}$$

where $\ln(S_{i,t})$ is the log value of intracity population mobility intensity in city i within time period t; $T_{i,t}$ is a dummy variable of groups, with the treatment group being 1 and the control group being 0; $R_{i,t}$ is the traffic control stage when Changsha started epidemic prevention and control (while Hunan Province started the first-level public health emergency response) on 24 January 2020, taking 1 during 24 January~5 February (on 6 February Hunan started progressive resumption of work and production and removed traffic prevention and control) or 0 during other time periods; $\omega_{0}$ is a constant; $\omega_{1}$ is the policy effect coefficient; $\omega_{3}$ is the treatment group coefficient; K is the coefficient of the interaction term $T_{i,t}\cdot R_{i,t}$, the one that calls for particular attention and that is solved by a regression model; and $\varepsilon_{i,t}$ is a disturbing term.

### 3.4. Model Parameter Calibration

**Figure 5:**
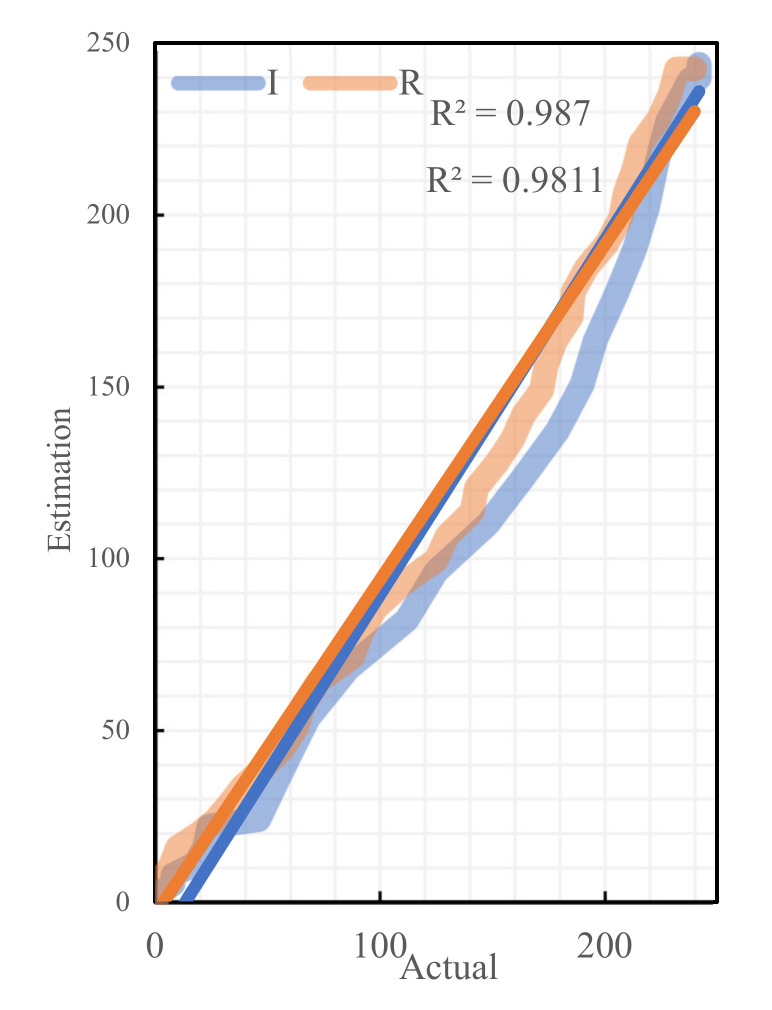
Regressive analysis of actual cases and theoretical cases.

**Figure 6:**
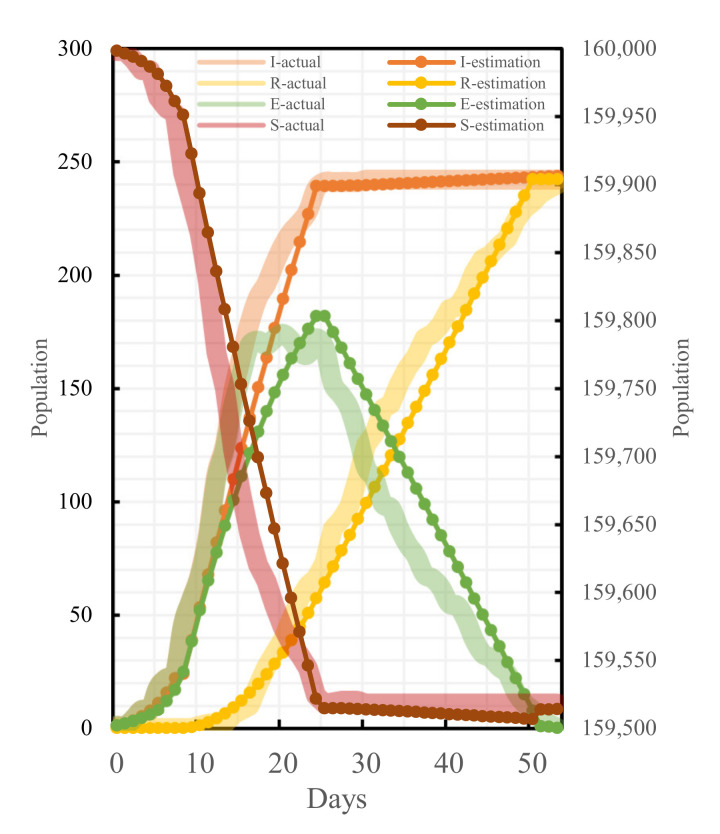
Fitting results of actual cases and theoretical cases.

To ensure the accuracy and the reasonability of the model result calculation, the parameters of the established model are calibrated in combination with the actual epidemic data of Changsha and available outcomes. The effective number of contacts r directly affects $\beta_{0}$. According to the literature [27], we set 10 scenarios such as r = (5,15) for $\beta_{0}$ estimation. The regression analysis was then performed on the estimated and the actual values of these 10 cases, and the results were significantly correlated, as shown in Table 4, indicating the robustness of the estimation method. Comparing the $\beta_{0}$ values under different r values, the Pearson correlation coefficient of infection rate at r = 15 was the closest to 1 and the best fit. To test the robustness and the sensitivity of the cure rate γ estimation results, we selected Changsha to exclude all other prefecture-level case data in Hunan Province to re-estimate the cure rate, and we found that there was still a significant correlation. They are shown in Figure 5. After determining $\beta_{0}$, the values of $\beta_{1}$, $\beta_{2}$, and $\beta_{3}$ were calculated by considering the administrative level of different policies as well as the time of promulgation and the scope of influence, respectively, as shown in Table 5 for the specific values. Meanwhile, the relative errors between some of the actual cases and theoretical cases were analyzed, as shown in Table 6. The fitting results of all parts are shown in Figure 6. Overall, this model can simulate the epidemic in Changsha.

## 4. Results

### 4.1. Epidemic Development Trend in the Absence of Traffic Control Policies

**Figure 7:**
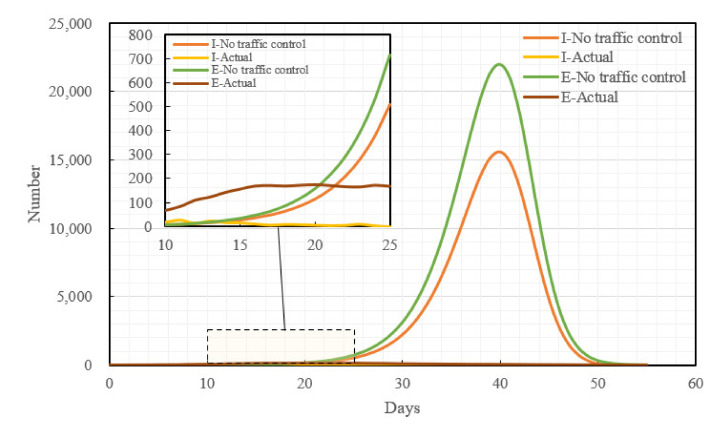
Comparison between the epidemic development trend without a traffic control policy and the actual epidemic development trend.

Had Changsha not taken traffic control measures at the initial stage of the outbreak of the epidemic, assuming that the virus had not mutated, the medical technology had been maintained at the same level as in the current stage, and allowing the epidemic to develop naturally, the epidemic trend in Changsha would have been as shown in Figure 7. Without having adopted the traffic control policies, both the peak value of the exposed (E-peak) and the peak value of the infective (I-peak) would have appeared on the 40th day of the epidemic, the E-peak would be 22,051 and the I-peak would be 15,606; compared with the real epidemic situation, the I-peak without having adopted the traffic control policies would have been 64 times the actual population of the infective, whereas the E-peak would have been 128 times the actual population of the exposed.

### 4.2. Epidemic Development Trend in the Presence of Traffic Control Policies

**Figure 8:**
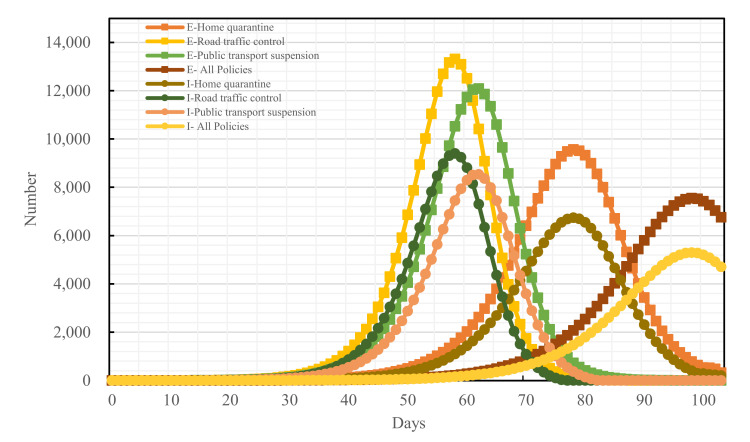
Epidemic development trends under all traffic control measures.

With the development of the epidemic, Changsha promulgated the three traffic control policies, home quarantine, road traffic control, and public transport suspension, on January 25th, 27th, and 28th, respectively. Assuming that the three traffic control policies were all implemented after Changsha started the first-level public health emergency response, compared with if none of the traffic control policies had been adopted, the I-peak dropped by 66.03%, the E-peak dropped by 65.70%, and the peak period of the infective population (I-population) was delayed by 58 days. Ignoring the superposed net effect of all measures, a simulation was performed for each of the traffic control measures. Compared with if none of the traffic control policies had been adopted, the home-quarantine policy decreased the I-peak and the E-peak by 56.81% and 56.76%, respectively, and it delayed the peak periods of the I-population and the exposed population (E-population) by 30 and 31 days, respectively; the implementation of the road traffic control policy decreased the I-peak and the E-peak by 39.72% and 39.51%, respectively, and it delayed both the peak periods of the I-population and the E-population by 18 days; the promulgation of the public transport suspension policy decreased the I-peak and the E-peak by 45.31% and 45.06%, respectively, and it delayed the peak periods of the I-population and the E-population by 22 and 23 days, respectively. Overall, all three policies can decrease the I-population and the E-population to varying degrees; the effect is optimal when all three traffic control policies are implemented simultaneously; the home-quarantine policy has the optimal effect among the three. The details are shown in Figure 8:

### 4.3. Blocking Effects of Different Traffic Control Policies on Epidemic Spread

**Figure 9:**
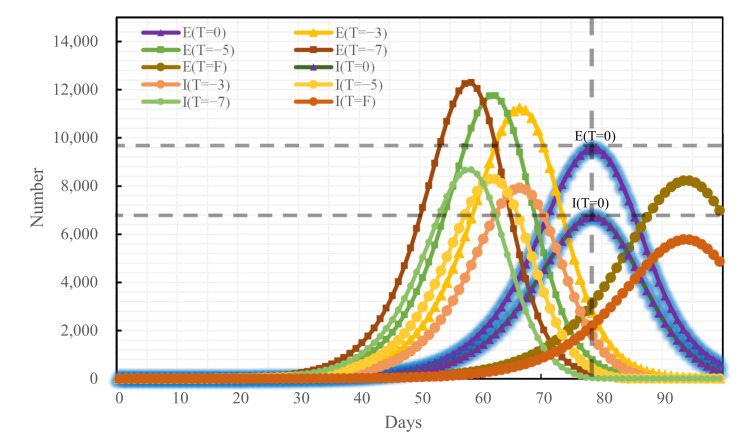
Epidemic trend under the home-quarantine policy promulgated at distinct time nodes.

**Figure 10:**
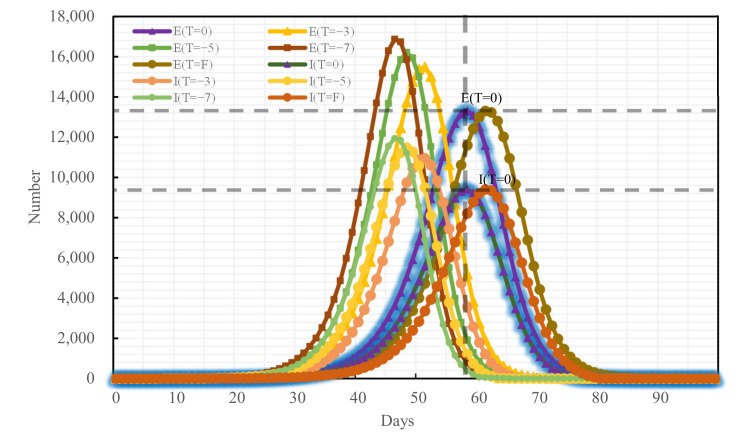
Epidemic trend under the road traffic control policy promulgated at distinct time nodes.

**Figure 11:**
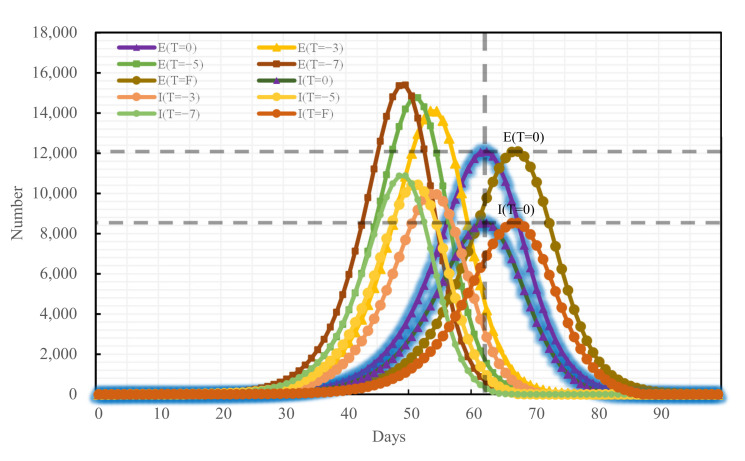
Epidemic trend under the public transport suspension policy promulgated at distinct time nodes.

To analyze the blocking effects of the implementation time of different traffic control policies on the epidemic spread across Changsha, the following four scenarios were set for each of the three traffic control policies: administering traffic control on the first day of the occurrence of medical cases, denoted as T = F, and delaying the time of actual implementation of traffic control policies by 3, 5, and 7 days, denoted as T = −3, T = −5, and T = −7, respectively, relative to the time of actual implementation of traffic control policies denoted as T = 0. The epidemic trends under all traffic control policies implemented at distinct points-in-time are shown in Figure 9, Figure 10 and Figure 11:

If the home-quarantine policy had been implemented at T = F, compared with the condition of T = 0 of this policy, the peak period would have been delayed by 18 days, while the E-peak and the I-peak would have decreased by 14.04% and 14.20%, respectively; compared with the condition of the no traffic control policy, the peak period was delayed by 54 days, while the E-peak and the I-peak decreased by 62.63% and 62.94%, respectively; if T = −3, T = −5, and T = −7, compared with the condition of T = 0 of this policy, the epidemic prevention and control would have become bad, and the E-peak and the I-peak would have increased by more than 17%. The later the policy was implemented, the larger the peak and the earlier the peak period would have arrived.

**Figure 12:**
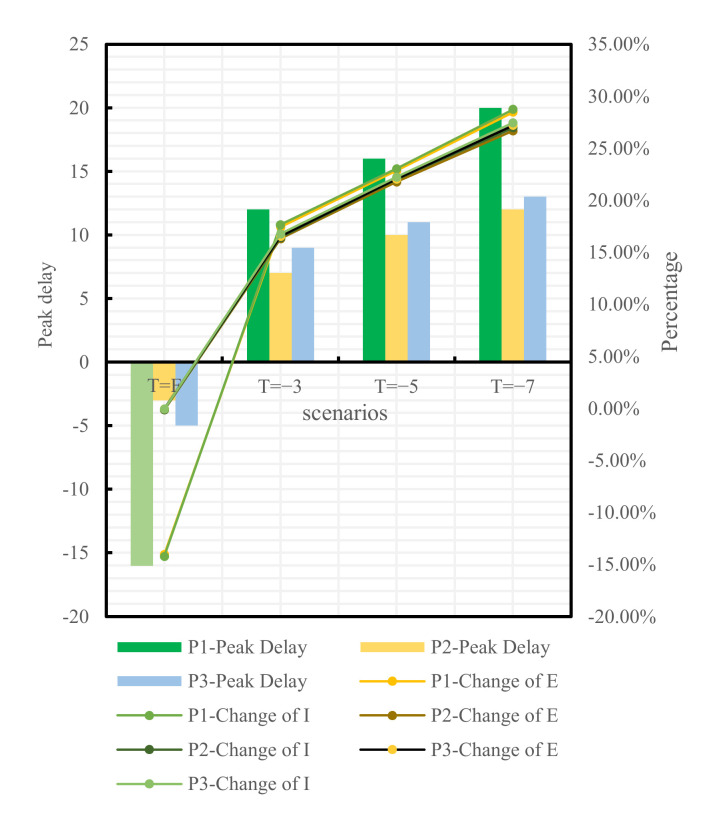
Comparison between T = F, −3, −5, −7, and T = 0 of different policies.

**Figure 13:**
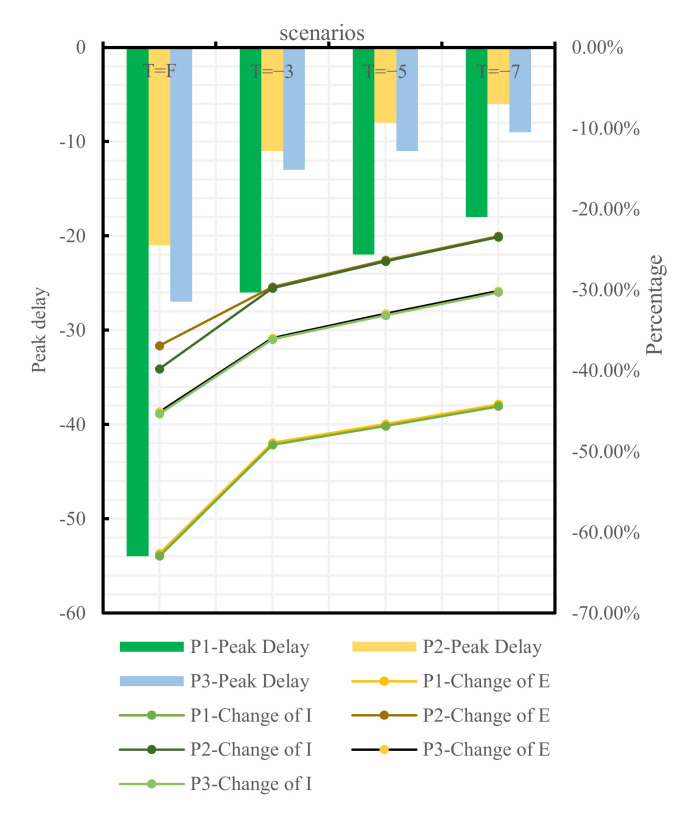
Comparison between T = F, −3, −5, −7 of different policies and no traffic control policy.

If the road traffic control policy and public transport suspension policy had been promulgated at T = F, compared with T = 0 of these policies, the peak period would have been delayed by 3 days and 5 days, respectively, while the E-peak would have decreased by 0.14% and 0.04%, respectively, and the I-peak would have decreased by 0.11% and 0.05%, respectively. The early implementation of these two measures would not have improved outbreak prevention and control. If T = −3, T = −5, T = −7, compared with the condition of T = 0 of these policies, the E-peak and the I-peak would have increased by more than 16%, as shown in Table 7 and in Figure 12 and Figure 13.

## 5. Discussions and Conclusions

### 5.1. Discussions

Enlightened by the classical SIR model and the research by scholars on the variant epidemic models pertinent to COVID-19, we have proposed a quantitative SEIR model considering the infectivity of the exposed for traffic control policies, designed the parameter of the infectious rate, included the policy effect parameter, and utilized the population migration data closely related to COVID-19 for calibration [32]. This model can provide a tool for preliminary decision-making effect evaluation for policymakers to make the most reasonable control policy. Plus, we believe the model will be useful for the effectiveness evaluation of policies in other regions after modifying the parameters. This modeling ideal can also provide a reference for the effectiveness evaluation of policies in other fields.

According to the evaluation results of the traffic control policies in Changsha, these policies can not only reduce the infective population but also delay the peak period effectively. This result is in general agreement with the conclusions obtained by Kraemer [33] and Bisanzio [34], which means effective physical isolation measures can mitigate the pressure on the local medical system, the logistics service, and on public governance. There exists a certain difference in the effects of the three preventive policies: home quarantine, road traffic control, and public transport suspension—with the home-quarantine policy having a superior effect to the other two and validating a related study done by Liu [35]. The reason is that the home-quarantine policy was implemented at an earlier time than the road traffic control and the public transport suspension policies and that the administrative grade and control sphere of the first policy’s promulgator was superior to the counterparts of the second and the third policies. On the other hand, this second fact also reminds decision-makers that in order to raise the influence of a policy to be implemented, a highly effective way is to implement it in advance and promote its promulgator’s administrative grade.

However, not all earlier implemented policies are more effective. From the results in Table 5 and Figure 11, adopting the road traffic control policy and the public transport suspension policy at the first case in Changsha would have resulted in a decrease of 0.14% and 0.04%, respectively, a limited improvement compared with T = 0. COVID-19 is prevented and controlled mainly by cutting off population mobility and aggregation [11], while road traffic control and public transport suspension only cut off the pathway of population mobility without preventing local population aggregation in substance [36]. However, home quarantine divides a community population into individual families and is a key approach to reduce the outward spread of cases and the chances of cross infections [37]. Therefore, home quarantine is most effective if implemented at the first case, which is highly consistent with the findings of Lai [38]. The variations of the peak value and the peak period timespan of confirmed cases under the home-quarantine policy would have been greater than those under the road traffic control and the public transport suspension policies if all policies had been delayed by 3, 5, and 7 days, respectively, showing that the home-quarantine policy has the highest sensitivity to implementation time. It is also important to note that the blocking measures of public transport suspension and home quarantine are closer in effect, possibly because both policies are controlled by the city government for the whole of Changsha. Both policies result in the loss of the ability of city residents to move quickly over short distances and a reduction in gathering time. Road traffic control differs from these two in that road traffic control takes a hierarchical approach. High- and medium-risk areas in Changsha are completely disrupted, while private cars in low-risk areas can pass normally with health code and trip code checks as well as body temperature tests. Therefore, there is still a small population movement, which may increase the risk of epidemic transmission and result in prevention and control that will be less effective than public transport suspension. Decision-makers can make a judgment on the epidemic in a city and select the most appropriate point in time to make the most appropriate traffic control policy to block the spread of COVID-19.

There are still many limitations to this study. Firstly, in the mathematical modeling, the group of asymptomatic infected persons was not considered in order to make the model more computationally convenient, which may lead to overestimation of the infection rate. Second, the application of the model is only applicable to concentrated outbreaks, not to localized episodic outbreaks, and further model improvement is needed for the evaluation of later regular traffic prevention and control policies. Finally, the setting of the scenario for the numerical simulation is too simple. Further precise settings are possible, e.g., in 1 daytime unit.

### 5.2. Conclusions

Based on the classical SIR model, this paper has reviewed the infectivity of the exposed, designed and improved the infectious rate by introducing the parameter of the traffic control policy effect, and built a quantitative SEIR model considering the infectivity of the exposed for traffic control policies. Taking Changsha as the study case and based on the big data from Baidu Qianxi, this paper has utilized the empirical model DID to calibrate the policy effect parameter of this city. Finally, the three traffic control policies implemented in Changsha—home quarantine, road traffic control, and public transport suspension—have been evaluated. The main conclusions drawn are as follows:

1. Based on the classical SIR model, a SEIR model considering the infectivity of the exposed for traffic control policy evaluation has been built. Compared with other traditional SIR models, this model has allowed for the infectivity signature of the exposed and designed the parameter of the infectious rate. This model can evaluate the blocking effects of traffic control policies on the spread of cases in a way.
2. According to the calculation results from the model, compared with the natural development state of the epidemic in Changsha, adopting traffic control policies has decreased the peak values of the infective and the exposed by 66.03% and 65.70% and delayed the peak period by 58 days. Among them, the home-quarantine policy is more significantly effective in decreasing the infective and the exposed populations in Changsha, and it can delay the peak period of the epidemic longer, compared to the road traffic control and the public transport suspension policies.
3. According to the results in different scenarios, the home-quarantine policy has higher time sensitivity than the road traffic control and the public transport suspension policies: the earlier this traffic control policy is implemented, the more significant its blocking effect on the spread of the epidemic. If simplex traffic control were the only need in an early stage, the home-quarantine policy would be the optimal choice.

In future work, we can consider modeling localized episodic COVID-19 outbreaks, which can include asymptomatic infected individuals as well as infected individuals with different symptom levels as the study population. Additionally, because of the extremely rapid mutation of COVID-19 strains, the characteristics of different strains can be modeled. What can be further studied is that we only discussed the effect of traffic control policies, it is also very meaningful to study other government interventions. Of course, in the future, a larger sample size of data can be obtained and deep learning methods can be used to improve the credibility of the model.

## Figures and Tables

**Table 1:** Related work on policy evaluation models.

Related Work	Modeling Method	Description	This Paper
[18]	Equation-based model SEIR	Consider “Quarantine” (Q), a SEIQR model was developed. Assessed the importance of social distancing and lockdown in changing human behavior. Changes in infection rates under different policies are not considered.	Infection rates were designed to consider the timeliness, scope of impact, and administrative level of policy enactment. Calibration of policy effect coefficients using population migration data based on the DID model. The effectiveness and the differences of the three traffic control policies in Changsha are discussed. The development process of the epidemic was simulated and the peak and peak periods were obtained for different policies.
[19]	Equation-based model SEIR	Consider “Asymptomatic” (A), “Isolated” (I), and “Hospitalized” (H), a SEAHIR model was developed. Assessed the impact of non-pharmaceutical measures. Not designed considering infection rates under different non-pharmaceutical measures.	
[20]	Equation-based model SIR	Consider “Dead” (D), a SIRD model was developed. Quantifying the importance of social distance for prevention and control. No consideration of the infectious properties of the exposed population.	
[21]	Equation-based model DID (Difference-in-Difference model)	Considering policy effects and population mobility coefficients on the base DID. Studied the impact of transportation policy on the flow of passengers on the ground drop. Only the final results are shown, not the development of COVID-19 outbreak.	
[22]	Equation-based model DID	The impact of different stages of transportation policies on population mobility in Changsha was studied. No in-depth study of the interdiction effect of traffic control policies on the outbreak.	
[23]	An equation-based econometric approach to empirically	Regression modeling of policy effects for different epidemic phases. Assessing the role of policies to limit population mobility and non-pharmaceutical interventions No prediction of outbreak development and peak.	

**Table 2:** Contents of all key measures.

Order	Date	Traffic Control Policy	Range of Control
1	25 January 2020	home quarantine	downtown Changsha
2	27 January 2020	road traffic control	high-risk and medium-risk areas
3	28 January 2020	public transport suspension	downtown Changsha

**Table 3:** Explanation of connotations of all parameters.

Parameter	Connotation	Parameter	Connotation
S(t)	size of subpopulation *S* at time *t*	r	number of the exposed to the susceptible per day
E(t)	size of subpopulation *E* at time *t*	$\beta_{0}$	initial infection probability after exposure to the exposed
I(t)	size of subpopulation *I* at time *t*	α	probability of the exposed subpopulation transmuting into the infective
R(t)	size of subpopulation *R* at time *t*	γ	cure rate
A	size of the resident population in downtown Changsha	θ	proportion of citywide effective active population

**Table 4:** The calculated $\beta_{0}$ values for different values of r.

r	$\beta_{0}$	R^2^	*adj*-R^2^	*p*	*Sig.*
5	0.049	0.907	0.899	0.808	0.000
6	0.048	0.927	0.918	0.820	0.000
7	0.046	0.937	0.929	0.826	0.000
8	0.045	0.945	0.937	0.831	0.000
9	0.043	0.954	0.947	0.836	0.000
10	0.041	0.960	0.952	0.840	0.000
11	0.040	0.968	0.961	0.844	0.000
12	0.039	0.974	0.967	0.848	0.000
13	0.037	0.980	0.973	0.852	0.000
14	0.036	0.985	0.975	0.855	0.000
15	0.035	0.991	0.981	0.858	0.000

**Table 5:** Values of all parameters.

Parameter Name	Value	Source	Notes
r	5~15 individuals	Literature [27]	The number of people with effective exposure varies by date and adjustment of prevention and control policies.
α	0.048~0.5	Literatures [28,29]	Taken as the reciprocal of the latent period, which lasts 2~21 days
θ	2%	Literature [30]	None
$\beta_{0}$	0.035	calculated by Formula (14)	None
γ	0.001	calculated by Formula (15)	None
K	−0.597 ***	calculated by Formula (16)	The numerical estimates have significant correlation
A	8,000,000	Changsha Bureau of Statistics [31]	resident population 10.048 million, including 8 million in urban area, throughout 2021
$\beta_{1}$	0.011	calculated by Formula (6)	infectious rate under the home-quarantine policy
$\beta_{2}$	0.019	calculated by Formula (6)	infectious rate under the road traffic control
$\beta_{3}$	0.017	calculated by Formula (6)	infectious rate under the public transport suspension policy

Descriptions: *** means *p* < 0.001.

**Table 6:** Relative error analysis of some data.

Date	I-Actual	I-Estimation	Relative Error	R-Actual	R-Estimation	Relative Error
25 February 2020	242	240	−0.63%	159	135	−15.29%
26 February 2020	242	241	−0.55%	164	142	−13.55%
27 February 2020	242	241	−0.48%	172	149	−13.45%
28 February 2020	242	241	−0.40%	174	156	−10.36%
29 February 2020	242	241	−0.32%	178	163	−8.37%
1 March 2020	242	241	−0.24%	185	170	−7.97%
2 March 2020	242	242	−0.16%	186	177	−4.62%

**Table 7:** Comparative analysis between epidemic trends under different policies.

Policy	Scenarios	E-Peak	I-Peak	Peak-Day	Comparison with T = 0			Comparison with No Policy		
					Peak Delay	Change of E	Change of I	Peak Delay	Change of E	Change of I
P1 ^1^	T = F	8227	5783	94	−16	−14.04%	−14.20%	−54	−62.63%	−62.94%
	T = 0	9571	6740	78	None	None	None	−38	−56.52%	−56.81%
	T = −3	11,240	7929	66	12	17.44%	17.64%	−26	−48.94%	−49.19%
	T = −5	11,757	8293	62	16	22.84%	23.04%	−22	−46.60%	−46.86%
	T = −7	12,296	8678	58	20	28.47%	28.75%	−18	−44.15%	−44.39%
P2 ^1^	T = F	13,298	9398	61	−3	−0.14%	−0.11%	−21	−36.90%	−39.78%
	T = 0	13,317	9408	58	None	None	None	−18	39.51%	−39.72%
	T = −3	15,488	10,952	51	7	16.30%	16.41%	−11	−29.65%	−29.82%
	T = −5	16,218	11,476	48	10	21.78%	21.98%	−8	−26.33%	−26.46%
	T = −7	16,873	11,946	46	12	26.70%	26.98%	−6	−23.36%	−23.45%
P3 ^1^	T = F	12,090	8531	67	−5	−0.04%	−0.05%	−27	−45.08%	−45.35%
	T = 0	12,095	8535	62	None	None	None	−22	−45.06%	−45.30%
	T = −3	14,096	9968	53	9	16.54%	16.79%	−13	−35.97%	−36.13%
	T = −5	14,758	10,431	51	11	22.02%	22.21%	−11	−32.96%	−33.16%
	T = −7	15,385	10,878	49	13	27.20%	27.45%	−9	−30.12%	−30.30%

^1^ The home-quarantine policy is denoted as P1, the road traffic control policy as P2, and the public transport suspension policy as P3.

## Data Availability

Not applicable.
